# Continuous Theta-Burst Stimulation in Children With High-Functioning Autism Spectrum Disorder and Typically Developing Children

**DOI:** 10.3389/fnint.2020.00013

**Published:** 2020-03-13

**Authors:** Ali Jannati, Gabrielle Block, Mary A. Ryan, Harper L. Kaye, Fae B. Kayarian, Shahid Bashir, Lindsay M. Oberman, Alvaro Pascual-Leone, Alexander Rotenberg

**Affiliations:** ^1^Neuromodulation Program and Division of Epilepsy and Clinical Neurophysiology, Department of Neurology, Boston Children’s Hospital, Harvard Medical School, Boston, MA, United States; ^2^Berenson-Allen Center for Noninvasive Brain Stimulation and Division of Cognitive Neurology, Department of Neurology, Beth Israel Deaconess Medical Center, Harvard Medical School, Boston, MA, United States; ^3^Neuroscience Center, King Fahad Specialist Hospital Dammam, Dammam, Saudi Arabia; ^4^Neuroplasticity and Autism Spectrum Disorder Program, Department of Psychiatry and Human Behavior, E. P. Bradley Hospital, Warren Alpert Medical School, Brown University, East Providence, RI, United States; ^5^Institut Guttman de Neurorehabilitació, Universitat Autónoma de Barcelona, Badalona, Spain

**Keywords:** transcranial magnetic stimulation, continuous theta-burst stimulation, plasticity, biomarker, autism spectrum disorder, *BDNF*

## Abstract

**Objectives**: A neurophysiologic biomarker for autism spectrum disorder (ASD) is highly desirable and can improve diagnosis, monitoring, and assessment of therapeutic response among children with ASD. We investigated the utility of continuous theta-burst stimulation (cTBS) applied to the motor cortex (M1) as a biomarker for children and adolescents with high-functioning (HF) ASD compared to their age- and gender-matched typically developing (TD) controls. We also compared the developmental trajectory of long-term depression- (LTD-) like plasticity in the two groups. Finally, we explored the influence of a common brain-derived neurotrophic factor *(BDNF)* polymorphism on cTBS aftereffects in a subset of the ASD group.

**Methods**: Twenty-nine children and adolescents (age range 10–16) in ASD (*n* = 11) and TD (*n* = 18) groups underwent M1 cTBS. Changes in MEP amplitude at 5–60 min post-cTBS and their cumulative measures in each group were calculated. We also assessed the relationship between age and maximum cTBS-induced MEP suppression (ΔMEP_Max_) in each group. Finally, we compared cTBS aftereffects in *BDNF* Val/Val (*n* = 4) and Val/Met (*n* = 4) ASD participants.

**Results**: Cumulative cTBS aftereffects were significantly more facilitatory in the ASD group than in the TD group (*P*_FDR_’s < 0.03). ΔMEP_Max_ was negatively correlated with age in the ASD group (*r* = −0.67, *P* = 0.025), but not in the TD group (*r* = −0.12, *P* = 0.65). Cumulative cTBS aftereffects were not significantly different between the two *BDNF* subgroups (*P*-values > 0.18).

**Conclusions**: The results support the utility of cTBS measures of cortical plasticity as a biomarker for children and adolescents with HF-ASD and an aberrant developmental trajectory of LTD-like plasticity in ASD.

## Introduction

Autism spectrum disorder (ASD) is characterized by social communication deficits and restricted, repetitive, and stereotyped behaviors and interests (American Psychiatric Association, 2013). Due to the large variability in the clinical phenotype of ASD and manifestation of symptoms over a range of ages in childhood, a clinical diagnosis of ASD can be challenging and is often not made until 3–5 years of age. For this reason, a neurophysiologic ASD biomarker is highly desirable, particularly for improving diagnostic specificity and for enabling metrics of therapeutic target-engagement and outcomes.

Aberrant synaptic plasticity in patients with ASD can be measured *in vivo* at the circuit level by transcranial magnetic stimulation (TMS; Huang et al., 2005; Hallett, 2007; Pascual-Leone et al., 2011). TMS enables focal noninvasive brain stimulation by electromagnetic induction (Barker et al., 1985; Kobayashi and Pascual-Leone, 2003; Hallett, 2007), to evoke or modulate neural activity in a given brain region or network (Valero-Cabré et al., 2017). When the recommended guidelines are followed (Rossi et al., 2009; Rossini et al., 2015), TMS is safe and well-tolerated, even in pediatric populations (Garvey and Gilbert, 2004; Rajapakse and Kirton, 2013; Hameed et al., 2017). TMS, when combined with electromyography (EMG), electroencephalography (EEG), or neuroimaging such as functional magnetic resonance imaging (fMRI) can quantify the extent of modulation of cortical reactivity induced by an intervention, providing an index of brain plasticity (Pascual-Leone et al., 2011).

Patterned repetitive TMS (rTMS) protocols in humans approximate experimental protocols that predictably induce long-term depression (LTD) and long-term potentiation (LTP) of synaptic strength in animal models (Huang et al., 2005, 2008). A form of rTMS termed continuous theta-burst stimulation (cTBS) consists of 50 Hz bursts of three TMS pulses repeated at 5 Hz for a total of 600 pulses over 40 s (Huang et al., 2005). Following cTBS of the primary motor cortex (M1), the average amplitude of motor evoked potentials (MEPs) induced by single TMS pulses is typically reduced by 25% for up to 50 min, before returning to pre-cTBS baseline (Wischnewski and Schutter, 2015). The cTBS-induced neuromodulatory effect has LTD-like characteristics (Cárdenas-Morales et al., 2011) and involves mechanisms of GABAergic and glutamatergic plasticity (Stagg et al., 2009; Trippe et al., 2009; Benali et al., 2011). Thus, cTBS aftereffects provide a neurophysiologic index of the mechanism of LTD-like cortical plasticity that is abnormal in patients with ASD (Pascual-Leone et al., 2005, 2011; Oberman et al., 2010).

Pursuant to cTBS (Oberman et al., 2012), adults with high-functioning (HF) ASD have greater and longer-lasting MEP suppression as compared to neurotypical (NT) controls, indicating an exaggerated, *hyperplastic*, response to patterned cortical stimulation. Additionally (Oberman et al., 2014), cTBS measures of plasticity among children and adolescents with HF-ASD demonstrate a positive linear relationship between age and the extent of cTBS-induced modulation. These findings reveal an age-related increase in LTD-like plasticity in childhood and adolescence.

We now extend the scope of previous cTBS studies in 10–16 years old children with high-functioning ASD addressing two questions: (1) are cTBS aftereffects different between ASD and typically developing (TD) groups? (i.e., is the cTBS biomarker adequate to distinguish children and adolescents with HF-ASD from age-matched TD controls?); and (2) does the developmental trajectory of cortical plasticity, as measured by cTBS aftereffects, differ between the two groups? (i.e., is there cortical dysmaturity in children with HF-ASD associated with delayed or aberrant maturation of LTD-like plasticity as measured by cTBS?). We also conduct pilot analysis on a subset of the ASD group, to test whether cTBS measures of plasticity were affected by a common single-nucleotide polymorphism (SNP) in the brain-derived neurotrophic factor (*BDNF*) gene, Val66Met, which has influences on rTMS measures of cortical plasticity in healthy subjects (Cheeran et al., 2008; Antal et al., 2010; Lee et al., 2013; Chang et al., 2014; Di Lazzaro et al., 2015; Fried et al., 2017; Jannati et al., 2017; Jannati et al., 2019).

## Materials and Methods

### Participants

Twenty-nine individuals participated in this study, which was approved by the local Institutional Review Board in accordance with the Declaration of Helsinki. All participants or their parents/legal guardians provided written informed consent/assent prior to enrollment and received age-appropriate monetary compensation in the form of a gift card upon completion. No participants endorsed TMS-specific contraindications (Rossi et al., 2009), and neurological examination was unremarkable for all participants enrolled. The two study populations were as follows: (1) high-functioning children with idiopathic ASD (*n* = 11; *ASD group*); and (2) neurotypical age- and gender-matched controls (*n* = 18; *TD group*). The TD group were originally recruited as part of a separate and unrelated study, and not for the purpose of comparing cTBS responses between TD and ASD children. Participants were recruited through local community advertisements, and local autism associations and clinics. All participants in the ASD group carried a prior clinical diagnosis made by a psychiatrist or clinical psychologist, met diagnostic criteria for ASD as defined by the *Diagnostic and Statistical Manual of Mental Disorders*, 5th edition (DSM-5^©^; American Psychiatric Association, 2013), and underwent independent neuropsychological assessment *via* the Autism Diagnostic Observation Schedule (ADOS; mean score = 10.82; SD = 3.28). Participants in the ASD group underwent a comprehensive neurological exam by a board-certified pediatric neurologist (Alexander Rotenberg, study M.D.) to confirm the absence of impaired gross or fine motor function. Participants in the TD group had no neurological or psychological disorder. Lastly, all participants were screened following published recommendations endorsed by the International Federation of Clinical Neurophysiology (see Table 1 for detailed demographic information).

**Table 1:** Demographics, neuropsychological measures, and medications for individual participants.

Group	Participant No.	Age	IQ	*BDNF*	Neuroactive medication	Comorbidities	ADOS score
ASD (*n* = 11)		*M* = 13.09 (*SD* = 1.97)	*M* = 103.55 (*SD* = 12.43)	4 Val/Val; 4 Val/Met	10 Yes; 1 None	9 Yes; 2 None	*M* = 10.82 (*SD* = 3.28)
	AS1	10–15	106	Val/Val	Dexmethylphenidate; Sertraline	ADHD; Anxiety	11
	AS2	10–15	82	Val/Val	None	None	13
	AS3	10–15	100	Val/Met	Flouxetine	Depression	15
	AS4	10–15	100	Val/Met	Adderall	ADHD; Asthma	7
	AS5	10–15	121	Val/Val	Clonidine; Melatonin; Sertraline	Anxiety; Depression; Asthma	10
	AS6	10–15	100	Val/Met	Dexmethylphenidate; Melatonin; Fluoxetine	Anxiety; Depression	9
	AS7	10–15	100	Val/Met	Clonidine; Methylphenidate	ADHD	10
	AS8	15–20	124	Val/Val	Melatonin; Methylphenidate	ADHD; PTSD; Asthma	16
	AS9	10–15	91	-	Melatonin	None	14
	AS10	10–15	100	-	Flouxetine; Thyroid tablets	Depression; Hypothyroidism	7
	AS11	10–15	115	-	Dexmethylphenidate	ADHD	7
TD (*n* = 18)		*M* = 13.44 (*SD* = 1.85)			0 Yes; 18 None	0 Yes; 18 None	-
	TD1	10–15			None	None	N/A
	TD2	15–20			None	None	N/A
	TD3	10–15			None	None	N/A
	TD4	10–15			None	None	N/A
	TD5	10–15			None	None	N/A
	TD6	10–15			None	None	N/A
	TD7	15–20			None	None	N/A
	TD8	10–15			None	None	N/A
	TD9	10–15			None	None	N/A
	TD10	15–20			None	None	N/A
	TD11	10–15			None	None	N/A
	TD12	10–15			None	None	N/A
	TD13	10–15			None	None	N/A
	TD14	10–15			None	None	N/A
	TD15	10–15			None	None	N/A
	TD16	10–15			None	None	N/A
	TD17	10–15			None	None	N/A
	TD18	15–20			None	None	N/A

*Group averages are presented as means and standard deviations (in parentheses). IQ scores were estimated using the Abbreviated Battery of Stanford-Binet IV intelligence scale. IQ, BDNF, and handedness data were not available for the TD group. Age range (instead of individual age) and no individual gender information are provided to avoid the possibility of publishing personally identifiable data. Three participants in the ASD group did not complete *BDNF* genotyping. ADHD; attention-deficit/hyperactivity disorder; ADOS, Autism Diagnostic Observation Schedule; ASD, autism spectrum disorder; BDNF, brain- derived neurotrophic factor; Met, metionine; PTSD, posttraumatic stress disorder; TD, typically developing; Val, Valine*.

### Neuropsychological Testing

The ADOS (Lord et al., 2000), and the Abbreviated Battery of Stanford–Binet IV intelligence scale (Thorndike et al., 1986) were completed for the ASD group. IQ scores were obtained prior to enrollment only for children with ASD to ensure patients with intellectual disability or low-functioning ASD were excluded from enrollment. IQ testing was not performed for TD children, with the assumption that the IQ of TD children falls within the normal limits of the general pediatric population.

We limited the enrollment of our ASD participants to HF children for two reasons: (1) lack of established feasibility of TMS/cTBS procedures in children with LF ASD; and (2) to reduce the heterogeneity of our pool of children with ASD, which are by nature, a heterogeneous population. As such, the findings reported in this study may be specific only to children and adolescents with HF-ASD.

### Genetic Testing

Saliva samples from participants in the ASD group (*n* = 8) were used to assess *BDNF* Val66Met SNP. The remaining three participants in the ASD group did not provide consent for DNA sampling and were thus not included in this subset.

Aliquot (700 μl) extraction of genomic DNA was performed on saliva samples collected using the Oragene Discover OGR-250 Kit (DNA Genotek Inc., Ottawa, ON, Canada). DNA was extracted from samples using standard methodology and the prepIT•L2P reagent (DNA Genotek Inc, 2015). The following quality control metrics were performed on each sample: PicoGreen fluorometry for double-stranded DNA quantification, Nanodrop spectrophotometry as an estimate of sample purity using A260/A280 ratios and agarose gel electrophoresis for visualization of DNA integrity.

The rs6265 SNP of the *BDNF* gene was analyzed using a TaqMan single-tube genotyping assay, which uses polymerase chain reaction (PCR) amplification and a pair of fluorescent dye detectors that target the SNP. One fluorescent dye is attached to the detector that is a perfect match to the first allele and a different fluorescent dye is attached to the detector that is a perfect match to the second allele. During PCR, the polymerase releases the fluorescent probe into solution where it is detected using endpoint analysis in an Applied Biosystems Inc. (Foster City, CA, USA) 7900HT Real-Time instrument. Primers and probes were also obtained through Applied Biosystems.

Because DNA samples were not available for the TD group, and a difference in rs6265 SNP prevalence in the ASD and TD groups could give rise to a difference in cTBS responses exhibited by the two groups, we estimated the probability that two hypothetical ASD and TD groups with sample sizes of 11 and 18, respectively, would have significantly different *BDNF* Met−:Met+ ratios. The minor allele frequency of the rs6265 SNP in the admixed American population in The 1000 Genomes Project Consortium et al. (2015) is approximately 0.153, which translates to 71.79%, 25.88%, and 2.33% for the prevalence of *BDNF* Val/Val, Val/Met, and Met/Met genotypes, respectively. Based on a 0.718:0.282 prevalence ratio of Met−:Met+ genotypes, we then conducted separate Monte Carlo simulations (Rubinstein and Kroese, 2016), each with 10,000 iterations, to estimate the probability that either 1, 2, …, or 11 subjects in the ASD group, and either 1, 2, …, or 18 subjects in the TD group would have a *BDNF* Met− genotype. We then conducted separate Fisher’s exact tests for all possible combinations of numbers of *BDNF* Met− subjects in the two groups and identified the scenarios in which the Met−:Met+ ratio would be significantly different between the two groups. For each of those scenarios, we then calculated the joint probability of the two relevant events in the two groups. Finally, we summated the probabilities of all those mutually exclusive scenarios to obtain an estimate of the overall probability that two groups of 11 and 18 subjects randomly sampled from the admixed American population would be significantly different from one another in *BDNF* Met−:Met+ ratio.

### Transcranial Magnetic Stimulation

Participants were seated in a comfortable reclining chair with the right arm and hand in a natural pronated position. They were instructed to keep their right hand as still and relaxed as possible throughout the experiment. They were also monitored for drowsiness and were asked to keep their eyes open during the TMS application.

All TMS procedures followed the recommended guidelines endorsed by the International Federation of Clinical Neurophysiology (Rossi et al., 2009; Rossini et al., 2015). Single TMS pulses and cTBS were applied to the left M1 at 120% of individual resting motor threshold (RMT) and 80% of active motor threshold (AMT), respectively, as biphasic pulses with an anteroposterior–posteroanterior (AP-PA) induced current direction in the brain. All stimulation was delivered using a hand-held figure-of-eight coil (outer diameter: 70 mm) attached to a Magstim Rapid^2^ Plus^1^ (Magstim Company Limited, Whitland, UK) stimulator.

The coil was held tangentially to the participant’s head surface, with the handle pointing occipitally and positioned at 45° relative to the mid-sagittal axis of the participant’s head. The optimal spot for the maximal TMS-induced motor responses of the right first dorsal interosseous (FDI) muscle (“motor hotspot”) was localized. A Polaris infrared-optical tracking system (Northern Digital Inc., Waterloo, ON, Canada) and a frameless stereotactic neuronavigation system (Brainsight, Rogue Research Inc., Montreal, QC, Canada) with a brain MRI template were used to ensure consistent targeting throughout the experiment. Each participant’s head was registered to the MRI template using defined cranial landmarks to ensure the coil position and orientation was consistent with the MRI template (Ruohonen and Karhu, 2010).

Surface EMG electrodes were placed over the FDI belly (negative) and the first interphalangeal joint of the second finger (positive). The ground electrode was placed over the ipsilateral ulnar styloid process. The TMS system delivered triggered pulses that synchronized the TMS and EMG systems.

At the start of each TMS session the FDI motor hotspot was located per patient and individual RMT, defined as the lowest stimulation intensity necessary to elicit an MEP of ≥50 μV in at least five of 10 pulses from the relaxed right FDI, was obtained. To assess pre-cTBS cortico-motor reactivity, three blocks of 30 single TMS pulses in the ASD group, and two blocks of 20 single TMS pulses in the TD group, were applied to M1 with a 5–10-min inter-block interval, at a random 4–6-s inter-pulse interval, as done in previous studies (Pechmann et al., 2012; Gomes-Osman and Field-Fote, 2015; Davila-Pérez et al., 2018).

The different number of single TMS pulses administered at baseline (90 vs. 40) and in each post-cTBS block (30 vs. 20) in the two groups was due to site-specific approval of the experimental protocol utilized at each research site. This difference was unlikely, however, to give rise to differing baseline MEP amplitude estimates between the two groups, as recent studies have found applying at least 20 single TMS pulses yields a reliable estimate of MEP amplitude at a given time point (Chang et al., 2016; Goldsworthy et al., 2016). In each block, individual MEPs >2.5 SD from the mean were excluded. The mean (±SD) number of MEPs in total excluded from all blocks in each subject was 4.63 (± 1.6) and 2.22 (±1.4) in the ASD and TD groups, respectively. This means that even in occasional post-cTBS blocks in the ASD group in which one (or, rarely, two) MEPs were excluded, there were 19 (or, rarely, 18) pulses remaining in each block, which has been shown to yield estimates of MEP amplitude with excellent internal consistency (Chang et al., 2016). To ensure hand relaxation was maintained throughout the experiment, real-time EMG was monitored to ensure the pre-TMS EMG activity did not exceed ~100 μV, which is the amplitude typically considered to be discernible activity from background EMG (Stinear and Byblow, 2002; Sartori et al., 2013; Benussi et al., 2018). Participants were also monitored for drowsiness and were asked to keep their eyes open for the duration of the stimulation session.

Baseline MEP amplitude was calculated as the average of the peak-to-peak amplitude of MEPs in the three blocks. AMT was then assessed as the lowest intensity that elicited MEPs ≥200 μV in at least five of 10 pulses with the FDI slightly contracted. Live EMG was monitored during the AMT assessment to ensure consistent contraction between ~100–200 μV. After a 5-min break, during which participants were instructed to maintain hand relaxation to control the effects of voluntary hand movements on cTBS responses (Iezzi et al., 2008), cTBS was applied as 200 bursts of three pulses at 50 Hz, repeated at 200-ms intervals for 40 s (for a total of 600 pulses). Cortico-motor reactivity was reassessed at 5, 10, 20, 30, 40, 50, and 60 min post-cTBS (*T5*–*T60*).

### Statistical Analyses

Study data were collected and managed using Research Electronic Data Capture (REDCap) electronic data capture tools hosted at Beth Israel Deaconess Medical Center (Harris et al., 2009, 2019). MATLAB R2016b (The MathWorks, Natick, MA, USA) and Stata 13.1 (StataCorp., College Station, TX, USA) were used for data analyses and simulations. G*Power 3.1.9 (Faul et al., 2007) was used for power and sample-size calculations.

Data from each TMS visit included: (a) RMT and AMT, expressed as percentage of maximum stimulator output (MSO); (b) baseline MEP amplitude, calculated as the average of baseline MEP amplitude in three blocks of 30 single TMS pulses; and (c) percent change in the average amplitude of 30 MEPs at T5–T60 relative to baseline (%Δ) for each participant.

The Shapiro–Wilk found significant deviations in MEP amplitudes from the normal distribution. Thus, we first baseline-corrected each post-cTBS amplitude by dividing it by the average baseline MEP amplitude in that individual participant. We then natural log-transformed the baseline-corrected MEP amplitudes at each post-cTBS time point (Δ*MEP*, Nielsen, 1996a,b; Pasqualetti and Ferreri, 2011) and averaged them over participants separately for each group. The following measures were also calculated: maximum suppression of MEPs during 60 min post-cTBS (*ΔMEP*_Max_) and the *signed* area-under-the-curve (AUC) of ΔMEPs over T5–T10, T5–T20,…, and T5–T60 intervals. To calculate the ΔMEP_Max_ for each participant, we chose the post-cTBS block (T5–T60) in which the ΔMEP showed the maximum suppression relative to the baseline MEP amplitude. Cumulative AUCs of the ΔMEPs enable numerical integration of cTBS-induced changes in MEP amplitude over successively larger intervals following cTBS. Such measures are more robust to the large inter-and intra-individual variability of MEP amplitudes typically observed at individual time points post-cTBS (López-Alonso et al., 2014; Vernet et al., 2014; Vallence et al., 2015; Hordacre et al., 2017; Jannati et al., 2017, 2019) and can be advantageous in studies with smaller sample sizes.

Grand-average values for all cTBS measures were calculated separately for each time point in each group and were compared between the two groups using independent-samples *t*-tests. Similar analyses were conducted for the two small *BDNF* subgroups (*n* = 4 per subgroup) of the ASD group. We conducted a sample-size analysis based on the preliminary results from the *BDNF* subgroups in order to estimate the number of participants per *BDNF* subgroup required to detect a significant difference between the cumulative AUC measures of cTBS aftereffects over each interval. Comparisons of proportions were conducted using Fisher’s exact test. Pearson product-moment correlation coefficient was used to assess the relationship between ΔMEP_Max_ and age in each group. All analyses were two-tailed, and α and β levels were set to 0.05 and 0.80, respectively. False discovery rate (FDR; Benjamini and Yekutieli, 2001) was used to adjust the *P*-values for multiple testing.

To obtain a rough estimate on the extent to which our small sample sizes—combined with the interindividual variability of MEP changes in response to cTBS—resulted in reduced power, we conducted a *post hoc* power calculation for the whole sample over each post-cTBS interval (but see Hoenig and Heisey, 2001 for the limited usefulness of this approach) and a pre-hoc sample-size calculation for the analyses comparing cTBS responses in the two *BDNF* subgroups of the ASD group.

To control for the number of pre- and post-cTBS MEPs in the two groups, we selected a subset of data from the ASD group such that both the number of baseline MEPs and the number of MEPs in each post-cTBS block included in the analysis would be equal in the two groups. Out of the 90 baseline MEPs, we selected the last 40 MEPs before cTBS to calculate the baseline MEP amplitude for each subject in the ASD group. We also selected the 20 MEPs out of 30 MEPs in each post-cTBS block that centered around the time point of interest (10, 20,…, and 60 min post-cTBS), and then log-transformed the baseline-corrected MEPs and recalculated the cumulative AUC measures of cTBS aftereffects for the ASD group. To account for the occasional MEP amplitudes excluded from each block in the TD group that was >2.5 SD, we continued to exclude any MEP that had been excluded from each block in the original, larger ASD dataset. Finally, we compared those cumulative measures of cTBS aftereffects with the corresponding measures in the TD group, as described above.

Even though our post-cTBS MEP measures are already baseline-corrected, it is still possible that a difference in the absolute baseline MEP amplitude between the two groups contributes to a difference in cTBS aftereffects. Because, as reported below, we found a significant difference in baseline MEP amplitude between the two groups, we set out to create the largest ASD and TD subgroups that would have comparable baseline MEP amplitude, and then compared the cTBS aftereffects between those subgroups.

Because several subjects in the ASD group had comorbid attention-deficit/hyperactivity disorder (ADHD; Table 1), and in pediatric ADHD there is impaired GABA-mediated plasticity as measured by paired-pulse TMS (Dutra et al., 2016; Gilbert et al., 2019), we repeated the calculation of cumulative AUC measures of cTBS aftereffects and their comparison between the ASD and TD groups after excluding the five ASD subjects with a documented clinical diagnosis of ADHD.

### Side-Effect Monitoring

Immediately following the TMS session, a side-effects questionnaire was completed by the experimenter. Participants were asked to report whether they experienced any of the following side effects: headache, neck pain, scalp pain or irritation, difficulty hearing, thinking or concentrating, change in mood, or to report any other change or side effect they experienced. The experimenter also noted whether the participant experienced a syncopal event or seizure. If the participant reported any side effects following the stimulation, the severity and duration were documented.

## Results

The ASD and TD groups were comparable in age and sex ratio (*P*-values > 0.61). Demographics, neuropsychological measures, and medications for individual participants are presented in Table 1.

### cTBS Is Safe and Tolerable in Children

All participants tolerated cTBS and single-pulse stimulation without any serious adverse event. One participant reported mild scalp irritation (on the forehead underneath the headband holding the subject tracker of the neuronavigation system), which was resolved quickly without medication. No other adverse events were reported.

### cTBS Measures of Plasticity Differentiate Between ASD and TD Children

**Figure 1:**
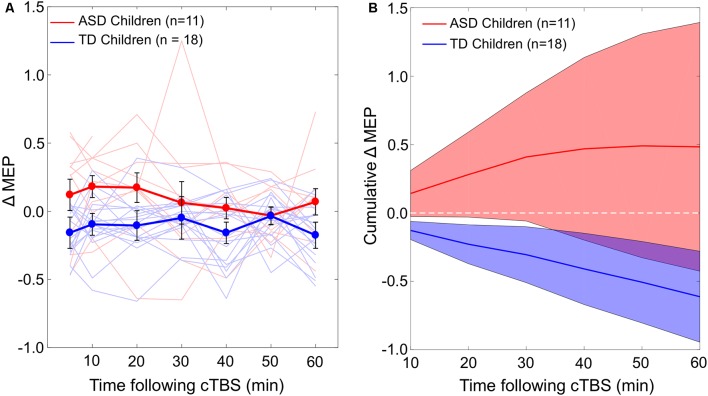
**(A)** Individual and grand-average change from baseline in MEP amplitude recorded from the right FDI muscle at 5–60 min following cTBS of the left motor cortex in ASD and TD groups. Error bars represent standard error of the mean. **(B)** Cumulative AUCs of the ΔMEPs and their 95% CI over T5–T10, T5–T20, …, and T5–T60 intervals for the two groups (the end time-point of each interval is labeled on the abscissa). The cumulative AUC measures were significantly more positive in the ASD group than in the TD group over all the T5–T10 to T5–T60 intervals (*P*_FDR_’s < 0.03). ASD, autism spectrum disorder; AUC, area-under-the-curve; CI, confidence interval; cTBS, continuous theta-burst stimulation; ΔMEP, natural log-transformed, baseline-corrected amplitude of motor evoked potentials; FDI, first dorsal interosseous; FDR, false discovery rate; MEP, motor evoked potential; TD, typically developing; *Tm*–*Tn*, over *m* to *n* minutes following cTBS.

The difference in cumulative AUC measures of cTBS aftereffects between the two groups was significant over all the intervals (*P*_FDR_’s < 0.03), indicating greater *facilitatory* response to cTBS in the ASD group relative to the TD group. Post-cTBS data from one participant in the ASD group were not obtained beyond T10 due to technical difficulties. Grand-average ΔMEPs at individual post-cTBS time points in the two groups are presented in Figure 1A. Cumulative AUCs of the ΔMEPs and their 95% confidence intervals (CI) over T5–T10, T5–T20,…, and T5–T60 intervals for the two groups are presented in Figure 1B.

The baseline MEP amplitude (mean ± SD) in the ASD group, 0.37 mV ± 0.27, was significantly smaller than in the TD group, 1.19 mV ± 0.41, *t*_(27)_ = 5.96, *P* < 0.001. The largest ASD and TD subgroups that would have a comparable baseline MEP amplitude consist of only five participants per subgroup. The baseline MEP amplitude [mean ± (SD)] in the two resulting subgroups with *n* = 5 are comparable: 0.62 mV (±0.17) in the ASD subgroup and 0.72 mV (±0.17) in the TD subgroup, *t*_(8)_ = 0.91, *P* = 0.39. There is no significant difference in cumulative AUC ΔMEP measures between the two subgroups over any of the post-cTBS intervals (*P*-values > 0.58).

The effect sizes based on the difference in cumulative AUC measure of cTBS aftereffects between the ASD and TD groups over T5–T10, T5–T20,…, and T5–T60 intervals are 0.85, 0.84, 0.79, 0.70, 0.65, and 0.65, respectively. Assuming two-tailed, independent-samples *t*-tests with *α* = 0.05, the *post hoc* power to detect a significant difference between the two groups are estimated as 57.2%, 56.2%, 51.2%, 42.2%, 37.4%, and 37.4%, respectively.

The Monte Carlo simulations find in a group of 11 subjects, the estimated probability that either 1, 2,…, or 11 subjects would have a *BDNF* Met—genotype is < 0.0001, 0.0002, 0.003, 0.012, 0.043, 0.115, 0.198, 0.266, 0.218, 0.113, and 0.027, respectively. In a group of 18 subjects, the estimated probability that either 1, 2,…, or 18 subjects would have a *BDNF* Met—genotype is <0.0001 (for 1–5 such subjects), 0.001, 0.003, 0.010, 0.031, 0.064, 0.120, 0.177, 0.208, 0.185, 0.127, 0.062, 0.019, and 0.002, respectively. After summating the probability of all scenarios in which a two-tailed Fisher’s exact test would find a significant difference in *BDNF* Met−:Met+ ratio between the ASD and TD groups, we obtain an overall probability of 0.0426.

After equalizing the number of pre- and post-cTBS MEPs in the two groups, the cumulative AUC measures of cTBS aftereffects do not change substantially compared to the measures obtained with the complete ASD dataset. The mean (±SD) cumulative AUC of ΔMEP over T5–T10, T5–T20,…, and T5–T60 intervals in this subset of data from the ASD group is 0.085 (±0.24), 0.18 (±0.45), 0.22 (±0.67), 0.24 (±0.80), 0.20 (± 0.92), and 0.14 (±1.06), respectively. These measures remain significantly more facilitatory in the ASD group than in the TD group over all the intervals from T5–T10 to T5–T50 (*P*_FDR_’s < 0.047), but not over T5–T60 (*P*_FDR_ = 0.053).

After excluding the five subjects in the ASD group who had comorbid ADHD, the cumulative AUC measures of cTBS aftereffects remain significantly more facilitatory in the ASD group compared to the TD group over all intervals (*P*_FDR_’s < 0.001).

### cTBS Aftereffects Have a Developmental Trajectory in Children with ASD

**Figure 2:**
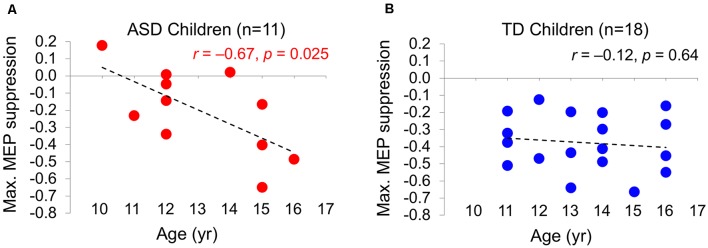
The relationship between the maximum cTBS-induced suppression in the natural log-transformed, baseline-corrected MEP amplitude (Max. MEP suppression) and age in ASD **(A)** and TD **(B)** groups. Max. MEP suppression was negatively correlated with age in the ASD group (*r* = –0.67, *P* = 0.025), but not in the TD group (*r* = –0.12, *P* = 0.65). Dashed lines represent the slopes of the linear regression fit. ASD, autism spectrum disorder; cTBS, continuous theta-burst stimulation; MEP, motor evoked potential; TD, typically developing.

ΔMEP_Max_ is correlated with age in the ASD group (*r* = –0.67, *P* = 0.025), but not in the TD group (*r* = –0.12, *P* = 0.65). The relationship between age and the maximum cTBS-induced suppression of MEPs (ΔMEP_Max_) during the first 60 min post-cTBS in the two groups are illustrated in Figure 2.

### *BDNF* and cTBS Measures of Plasticity in ASD

**Figure 3:**
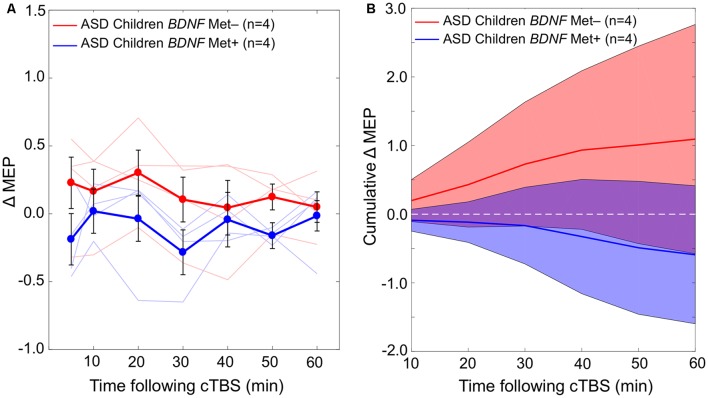
**(A)** Changes in individual and grand-average MEPs recorded from the right FDI muscle at 5–60 min following cTBS of the left primary motor cortex in *BDNF* Met− (Val/Val) and Met+ (Val/Met) subgroups of participants with ASD. Error bars represent standard error of the mean.** (B)** Cumulative AUCs of the ΔMEPs and their 95% CI over T5–T10, T5–T20, …, and T5–T60 intervals for the two *BDNF* subgroups (the end time-point of each interval is labeled on the abscissa). The cumulative AUC measures were not significantly different between the two *BDNF* subgroups over any of the intervals (*P*-values > 0.18). ASD, autism spectrum disorder; AUC, area-under-the-curve; *BDNF*, brain-derived neurotrophic factor; CI, confidence interval; cTBS, continuous theta-burst stimulation; ΔMEP, natural log-transformed, baseline-corrected amplitude of motor evoked potentials; FDI, first dorsal interosseous; FDR, false discovery rate; MEP, motor evoked potential; Met, methionine; TD, typically developing; *Tm*–*Tn*, over *m* to *n* minutes following cTBS; Val, valine.

The difference in cumulative AUC measures of cTBS aftereffects between the two *BDNF* subgroups (Val/Val and Val/Met) of the ASD group is not statistically significant (*P*-values > 0.08). Grand-average ΔMEPs at individual post-cTBS time points in the two *BDNF* subgroups are presented in Figure 3A. Cumulative AUCs of the ΔMEPs and their 95% CI over T0–T10, T0–T20, …, and T0–T60 intervals for the two *BDNF* subgroups are presented in Figure 3B.

Based on the results from eight participants with available *BDNF* data, the effect size based on the difference in cumulative AUC measure of cTBS aftereffects over each interval ranges from 0.78 to 0.87. Assuming a 1:1 ratio between the two subgroups, the sample size in each *BDNF* subgroup required to detect those effect sizes with 80% power ranges from 27 to 22, respectively.

## Discussion

We find, considering the caveats and confounders discussed below, the responses to M1 cTBS can differentiate between 10–16 years old children with HF-ASD and their age-, gender, and IQ-matched TD children. This is due to more-facilitatory cTBS aftereffects in MEPs in the ASD group relative to the TD group. We argue this difference is not likely to be due to potential confounds such as differences in the number of pre- and post-cTBS MEPs, *BDNF* Val66Met SNP, ADHD comorbidity, or neuroactive medications between the two groups. Moreover, we report an age-related increase in the maximum cTBS-induced suppression of MEPs in the ASD group, but not in the TD group, suggesting a dysmaturity in LTD-like plasticity in children with ASD. These results indicate the importance of further investigations of the utility of M1 cTBS as a potential physiologic biomarker for children and adolescents with HF-ASD.

### TMS Safety and Tolerability in Children

All participants tolerated the stimulation, and only one participant reported a minor scalp irritation that resolved quickly without medication. The present study adds to the growing literature documenting the safety and tolerability of rTMS in children and in individuals with ASD (Garvey and Gilbert, 2004; Frye et al., 2008; Croarkin et al., 2011; Oberman et al., 2012, 2014, 2016; Wu et al., 2012; Rajapakse and Kirton, 2013; Hong et al., 2015; Pedapati et al., 2015; Hameed et al., 2017).

### cTBS as a Biomarker for Children and Adolescents With ASD

We find that the pattern of cTBS aftereffects during the first 60 min post-cTBS successfully differentiates between the ASD and TD groups. Namely, the ASD group shows significantly more facilitatory responses to cTBS than the TD group throughout the assessed post-cTBS interval (Figure 1). Notably, this pattern of results is not driven by one or two particular post-cTBS time points, which can be prone to the large inter- and intra-individual variability in cTBS responses observed in adults (López-Alonso et al., 2014; Vernet et al., 2014; Vallence et al., 2015; Hordacre et al., 2017; Jannati et al., 2017, 2019).

The finding that the ASD group exhibits a distinct pattern of cTBS from their TD counterparts indicates the potential utility of M1 cTBS as a biomarker for children and adolescents with HF-ASD. Moreover, considering the involvement of GABAergic synaptic transmission in cTBS aftereffects (Stagg et al., 2009; Trippe et al., 2009), the facilitatory (rather than inhibitory) responses to cTBS in the ASD group further supports the notion of GABAergic dysfunction in ASD (LeBlanc and Fagiolini, 2011; Ben-Ari et al., 2012; Coghlan et al., 2012). The effect of GABAergic transmission during typical development shifts from excitatory to inhibitory through sequential activation of chloride (Cl^−^) cotransporters NKCC1 and KCC2 and *via* age-dependent reduction of intracellular Cl^−^ concentration (Yamada et al., 2004; Ben-Ari et al., 2007). Rodent ASD models indicate a delayed shift of GABA activity from excitatory to inhibitory, which can be restored behaviorally and electrophysiologically by *in utero* administration of the NKCC1 inhibitor bumetanide (Tyzio et al., 2014). Similarly, bumetanide treatment may mitigate core ASD symptoms in children and adolescents (Lemonnier et al., 2017). We thus suggest that cTBS measures of M1 plasticity in ASD can be used to assess baseline cortico-motor reactivity, probe-target engagement, and monitor therapeutic response to experimental pharmacotherapy (e.g., bumetanide; Lemonnier et al., 2017) and, potentially, future rTMS treatments for ASD (Cole et al., 2019). Moreover, differential cTBS responses within the pediatric ASD population can form the physiologic basis for a clinical endophenotype that improves classification and understanding of the pathophysiology of ASD.

The more-facilitatory response to cTBS in the ASD group relative to the TD group is consistent with the results of previous studies that have found impaired LTP-like changes in MEPs in individuals with ASD by paired associative stimulation (PAS; Jung et al., 2013) and iTBS (Oberman et al., 2012; Pedapati et al., 2016). Given that cTBS also likely engages GABAergic mechanisms, our results are also consistent with related findings that employ other TMS-derived biomarkers. For instance, GABA_A_ergic activity, as measured by short-interval intracortical cortical inhibition (SICI), was associated with a delay in language acquisition in adults with ASD (Enticott et al., 2013).

### Present and Anticipated Confounders

Although the post-cTBS measures reported in the present study are already adjusted for baseline MEP amplitude at the individual level, it is possible that a difference in the baseline MEP amplitude at the group level contributes to the observed differences in cTBS aftereffects between ASD and TD groups. The finding that the baseline MEP amplitude is significantly smaller in the ASD group than in the TD group can be either due to chance because of the small sample sizes or because of a real difference in input-output characteristics of MEPs in the two groups (Goetz et al., 2014). Because the largest ASD and TD subgroups with a comparable baseline MEP amplitude consist of only five participants, the present sample does not allow for a robust assessment of the effect of group-level baseline MEP amplitude on cTBS aftereffects in the two groups. Larger samples in future studies aimed at comparing rTMS responses between ASD and control populations can address this limitation by ensuring comparable baseline MEP amplitudes at the group level between the two groups.

Our analysis controlling for the number of baseline and post-cTBS MEPs shows that the differences in cumulative measures of cTBS aftereffects, at least from T5–T10 to T5–T50, are not due to differences in the number of baseline MEPs (90 vs. 40) or the number of MEPs in each post-cTBS block (30 vs. 20) between the ASD and TD groups.

One issue that needs to be considered in comparing cTBS responses between the ASD and TD groups is the potential effects of neuroactive medications on cTBS responses. It is plausible that at least some of those medications influence the pattern of cTBS aftereffects in ASD participants and thus the difference between the two groups. There is, however, considerable variability in the type of those medications received by our ASD participants, which makes it unlikely that all or a majority of them have a similar effect on the plasticity mechanisms indexed by cTBS aftereffects. To maintain the external validity of the findings of studies aimed at developing biomarkers or therapeutics for the ASD population, it is necessary to include patients who are under treatment by neuroactive medications prescribed for common comorbidities such as depression, anxiety, and ADHD.

Another issue in comparing cTBS responses between the ASD and TD groups is the possibility that there is a significant difference in *BDNF* Met−:Met+ ratio between the two groups. Such difference can give rise to an observed difference in cTBS responses between the two groups that are not necessarily associated with an ASD diagnosis but with the composition of *BDNF* genotypes in the two groups. Our simulations, however, do not find such a possibility to be very likely in the present study. We find, assuming random sampling from the admixed American population, there is a ~4.3% chance that two groups of 11 and 18 subjects are significantly different from one another in the *BDNF* Met−:Met+ ratio.

Potential confound due to psychiatric comorbidities in the ASD group is another factor that can mediate the difference in cTBS responses between the two groups. One common comorbidity in ASD is ADHD (Craig et al., 2015), in which abnormal GABA-mediated plasticity measured by paired-pulse TMS has been observed (Dutra et al., 2016; Gilbert et al., 2019). After excluding five subjects in the ASD group with documented ADHD comorbidity, we still find significantly greater cumulative facilitatory aftereffects of cTBS in the ASD-without-ADHD subgroup than in the TD group across all post-cTBS intervals. These results show that the observed differences between the whole ASD group and the TD group cannot not be due to the effects of ADHD comorbidity on cTBS responses. In general, nonetheless, in studies comparing plasticity responses of ASD and control populations, the potential effects of common psychiatric comorbidities such as depression, anxiety, and ADHD on TMS measures of plasticity, should be considered.

Interestingly, the overall pattern of cTBS responses in the ASD group in the present study is not necessarily what one would expect based on previous results. Namely, a previous cTBS study from our group (Oberman et al., 2014) found only one-third of children and adolescents with ASD showed facilitatory responses to cTBS. This difference in results can be due to several factors:

(1) The large inter-individual variability in response to cTBS among healthy adults is now well-established (Hamada et al., 2013; Goldsworthy et al., 2014; López-Alonso et al., 2014; Nettekoven et al., 2015; Vallence et al., 2015; Hordacre et al., 2017; Jannati et al., 2017). A similar degree of variability in cTBS responses in TD children and among clinical pediatric populations such as children with ASD is reasonable to expect. By mere virtue of random sampling from a large spectrum of responses, such variability can give rise to seemingly inconsistent results in small sample sizes that may not capture the entire gamut of cTBS responses in healthy and clinical populations. In fact, our *post hoc* power calculation for the whole sample indicates that the present study is underpowered, especially beyond the T5–T20 interval. Larger sample sizes would perhaps have resulted in more-robust differences, and/or over longer intervals, between the two groups. We thus anticipate that this power analysis will enable the design of future studies with larger sample sizes that are required to confirm and extend the present results.
(2) The difference in the proportion of participants with *BDNF* Val/Met genotype, which has been shown to influence cTBS and other rTMS measures of neuroplasticity (Cheeran et al., 2008; Antal et al., 2010; Lee et al., 2013; Chang et al., 2014; Di Lazzaro et al., 2015; Fried et al., 2017; Jannati et al., 2017, 2019) may have contributed to the different results.
(3) Differences in other demographic, neuropsychological, and genetic factors as well as neuroactive medications received by the participants in the ASD group could have given rise to the different patterns of cTBS response in the two studies.

These potential confounds underscore the need for replication of present findings in future cTBS studies with large samples of children and adolescents with ASD in order to overcome, or control for, some of these factors. Another important reason for replicating the present findings is to assess the test-retest reliability of M1 cTBS aftereffects in both HF-ASD and TD children. This is underscored by the recent findings in healthy adults that indicate low-to-moderate reliability of most cTBS aftereffects (Jannati et al., 2019).

Regarding potential selection and outcome biases in recruiting the participants in the TD group, it should be noted that because the TD subjects were recruited as part of an unrelated study—and not for the purpose of comparing their cTBS responses with those of ASD children—such biases did not play a role in recruiting the TD subjects.

### Developmental Trajectory of cTBS Responses in ASD

Consistent with the previously reported age-related increase in the duration of cTBS-induced modulation in children and adolescents with ASD (Oberman et al., 2014), we find an age-related increase in the maximum cTBS-induced suppression of MEP during 60 min post-cTBS in the ASD group, but not in the TD group. Namely, older participants with ASD tend to exhibit greater cTBS-induced LTD-like plasticity than younger participants with ASD, whereas there is no such developmental trajectory in the TD group (at least in the age range of 11–16). Caution should be exercised in interpreting these correlations, however, because of the short dynamic range of age in the two groups. Assuming these results are confirmed in larger studies in the future and over wider age ranges, they suggest a dysmaturity in LTD-like plasticity as measured by cTBS in children with ASD, perhaps arising from a dysfunction in the shift of GABAergic activity from excitatory to inhibitory. Since GABA_A_-receptor activity is involved in generating the cTBS aftereffects, such dysfunction would cause the ASD participants to achieve greater inhibitory cTBS responses as they grow older. In contrast, because the GABAergic shift presumably occurs earlier in TD children, they may achieve greater cTBS-induced inhibition at a younger age and then plateau at older ages. These results hint at the utility of cTBS measures of plasticity as longitudinal tools for monitoring the development of cortical plasticity and/or gradual response to potential treatments among children and adolescents with ASD. Because the slope of such developmental trajectory is likely to vary across individuals with ASD, a substantial number of subjects at any given age may be necessary to obtain robust “growth curves” for cortical plasticity in pediatric ASD populations.

### BDNF Polymorphism and cTBS Aftereffects in ASD

The role of *BDNF* Val66Met SNP in influencing rTMS plasticity measures in adults has been investigated in several studies (Cheeran et al., 2008; Antal et al., 2010; Lee et al., 2013; Chang et al., 2014; Di Lazzaro et al., 2015; Fried et al., 2017; Jannati et al., 2017, 2019). *BDNF* Met carrier status is known to be associated with impaired N-Methyl-D-aspartate-(NMDA)-dependent LTD (Woo et al., 2005), aberrant GABAergic synaptic transmission (Abidin et al., 2008), reduced cTBS-induced inhibition of MEPs (Chung et al., 2016), and, in some cases, paradoxical cTBS-induced facilitation of MEPs (Gentner et al., 2008; Goldsworthy et al., 2012; Hellriegel et al., 2012; Brownjohn et al., 2014; Jannati et al., 2017, 2019). In contrast with these results, the *BDNF* Met+ children with ASD in the present study exhibit a numerically more-*inhibitory* response than the *BDNF* Met− children at all individual post-cTBS time points, even though the difference between two subgroups is not statistically significant. Because of the small sample sizes, it is difficult to infer whether the seemingly opposite effect of *BDNF* Met carrier status on cTBS response in children with ASD compared to adults is due to: (1) sampling error arising from small sample sizes; or (2) a dysfunction in GABAergic shift that causes the *BDNF* SNP to have an opposite influence on cTBS aftereffects in children with ASD compared to healthy individuals.

## Conclusion

Considering the discussed limitations and potential confounders, cTBS-derived metrics may enable practical and safe physiologic biomarkers in pediatric ASD. Given that such measures can be applied repeatedly to individuals, our data also point to prospects for probing developmentally regulated features of cortical plasticity in ASD and perhaps other neurodevelopmental disorders. Because of its high tolerability by patients with ASD, cTBS offers an opportunity to study the mechanisms and alterations of neural plasticity in the ASD population. These proof-of-principle findings in the motor cortex can be followed in future studies through extra motor stimulation in TMS-EEG or similar protocols.

## Data Availability Statement

The datasets generated for this study are available on request to the corresponding author.

## Ethics Statement

This study was approved by the Institutional Review Boards at Boston Children’s Hospital and King Saud University, where the research took place, in accordance with the Declaration of Helsinki. All participants provided written informed consent/assent prior to enrollment and received monetary compensation upon completion.

## Author Contributions

AJ, LO, AP-L, and AR conceived and designed the study. AJ, GB, MR, HK, FK, SB, and LO collected the data. AJ analyzed the data and drafted the manuscript. AJ, AP-L, and AR interpreted the data. All authors revised the manuscript, approved the final version, and agreed to be accountable for the content of the work.

## Conflict of Interest

AR is a founder and advisor for Neuromotion, serves on the medical advisory board or has consulted for Cavion, Epihunter, Gamify, NeuroRex, Roche, Otsuka, and is listed as an inventor on a patent related to the integration of TMS and EEG. AP-L serves on the scientific advisory boards for Neuronix, Starlab Neuroscience, Neuroelectrics, Constant Therapy, Cognito, NovaVision, and Neosync; and is listed as an inventor on several issued and pending patents on the real-time integration of TMS with EEG and MRI. The remaining authors declare that the research was conducted in the absence of any commercial or financial relationships that could be construed as a potential conflict of interest.

## Abbreviations

ADHD: attention-deficit/hyperactivity disorder
AMT: active motor threshold
*BDNF*: brain-derived neurotrophic factor
cTBS: continuous theta-burst stimulation
ΔMEP: natural log-transformed, baseline-corrected amplitude of motor evoked potentials
EEG: electroencephalography
EMG: electromyography
FDR: false discovery rate
fMRI: functional magnetic resonance imaging
GABA: gamma-aminobutyric acid
HF: high-functioning
IQ: intelligence quotient
iTBS: intermittent theta-burst stimulation
LF: low-functioning
LTD: long-term depression
LTP: long-term potentiation
MEP: motor evoked potential
Met: metionine
PAS: paired associative stimulation
PCR: polymerase chain reaction
RMT: resting motor threshold
rTMS: repetitive transcranial magnetic stimulation
SICI: short-interval intracortical cortical inhibition
SNP: single-nucleotide polymorphism
TMS: transcranial magnetic stimulation
Tn: at *n* minutes post-cTBS
Val: valine
%Δ: percent change from the baseline.
